# Optimizing Druggability through Liposomal Formulations: New Approaches to an Old Concept

**DOI:** 10.5402/2012/738432

**Published:** 2012-02-09

**Authors:** Dimitrios Bitounis, Raphaelle Fanciullino, Athanassios Iliadis, Joseph Ciccolini

**Affiliations:** UMR 911 CRO2, Pharmacokinetics Laboratory, Aix-Marseille University, 13385 Marseille, France

## Abstract

Developing innovative delivery strategies remains an ongoing task to improve both efficacy and safety of drug-based therapy. Nanomedicine is now a promising field of investigation, rising high expectancies for treating various diseases such as malignancies. Putting drugs into liposome is an old story that started in the late 1960s. Because of the near-total biocompatibility of their lipidic bilayer, liposomes are less concerned with the safety issue related to the possible long-term accumulation in the body of most nanoobjects currently developed in nanomedicine. Additionally, novel techniques and recent efforts to achieve better stability (e.g., through sheddable coating), combined with a higher selectivity towards target cells (e.g., by anchoring monoclonal antibodies or incorporating phage fusion protein), make new liposomal drugs an attractive and challenging opportunity to improve clinical outcome in a variety of disease. This review covers the physicochemistry of liposomes and the recent technical improvements in the preparation of liposome-encapsulated drugs in regard to the scientific and medical stakes.

## 1. Introduction

**Figure 1:**
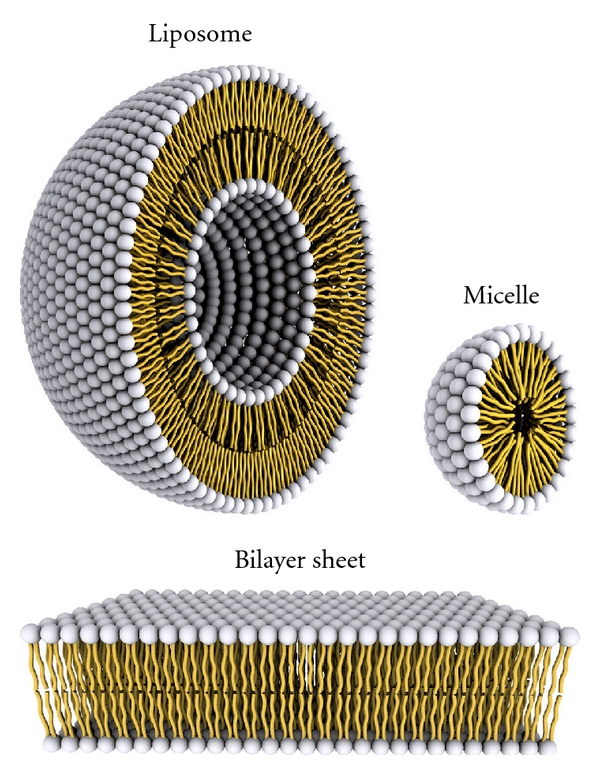
Aspects of liposomes and micelles. A representation of the steric organization of a liposome (left) and a micelle (right). Liposomes have a lipidic bilayer (bottom) whereas micelles are constructed only by one lipid layer that has its apolar section turned inwards while its polar heads interact with the environment. As a result, the enclosed space in micelles is much more confined to that available in liposomes.

Liposomes are nearly spherical, microparticulate, multilamellar or unilamellar bilayer vesicles made from lipids alternating with aqueous sections [1]. Their biochemical structure is very much similar to that of normal human cellular membranes. They also bear resemblance to micelles, although there are some key differences between them (Figure 1). They were first discovered by Dr Alec D. Bangham in 1961 at Babraham University of Cambridge [2].

 Because of the aforementioned similarity to natural components as well as their ability to enfold various substances, scientists hypothesized that liposomes complied with the requirements of an almost ideal drug carrier system. So, for the last 40 years liposomes have been studied thoroughly and are actually celebrated for their biological and technological advantages as effective carriers for biologically active substances, both in vitro and in vivo. Naturally, they continue to constitute a field of intense research and are considered to be the best drug carrier system known yet. Notable progress has been made during the last decade and various biomedical applications of liposomes have already been approved for public use or are on the verge of commercialization [3].

## 2. General Description

All liposomes have in common a compartmental structure which gives them the ability to function as storage and carrier systems for various substances. The use of liposomes as carrier systems is based on the fact that all liposomal content is protected against naturally occurring phenomena, such as enzymic degradation and immunologic and chemical inactivation. When the desired molecules are imported to the liposomes, at least one interjected lipidic layer insulates them from their environment. Other than that, the lipidic composition of the liposomal membranes guarantees their biocompatibility and biodegradability [4]. Last but not least, liposomal formulation allows for poorly soluble lipophilic and amphiphilic drugs to be better solubilized in aqueous solutions [5]. To summarize, liposomes can store, protect, and transfer substantial quantities of medicines while being well tolerated by the receiving organism. These unique traits provide for an upgraded biopharmaceutical profile through reduced toxicity and favourable pharmacokinetic behaviour and an improved therapeutic index in comparison to the free-form drug.

## 3. Physiochemistry of Liposomes

The efficacy of liposomes as a colloidal storage and carrier system for biologically drastic substances greatly depends on the physiochemical properties of their membranes and the nature of the enclosed agent. The former include their size, surface charge, lipidic organization, and chemical constitution, among others [6]. Hereinafter follows a generalized presentation of the physical and chemical traits of liposomes.

### 3.1. Chemical Traits

**Figure 2:**
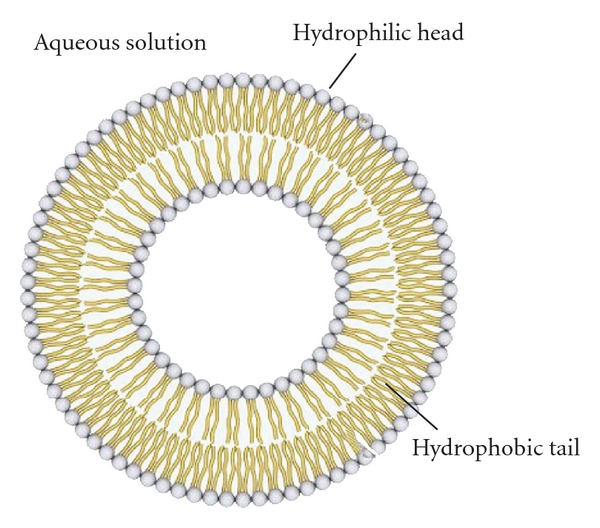
The fundamental organization of liposomes. In this figure one can observe the fundamental organization of liposomes with one bilayer and the direction that phospholipids adopt in order to form it.

Liposomes are composed of lipids. Lipids are amphiphile biomolecules that have either a charged or neutral polar head and at least one hydrophobic aliphatic chain. They are generally immiscible to aqueous solutions but very soluble to organic solvents. Although there are many types of lipids, liposomes are mainly consisted of phospholipids that have a hydrophilic head and two apolar hydrophobic chains (Figure 2). When dispersed in aqueous solutions, their steric organization aims to minimize the interactions between the hydrophobic chains and water molecules, thus spontaneously forms bilayer membranes, the liposomes [7]. Inside these membranes, ions or molecules can be encapsulated, provided that they are present during the formulation process. The final arrangement of lipids depends on their concentration, temperature, and geometric form.

#### 3.1.1. Anatomy of a Phospholipid

**Figure 3:**
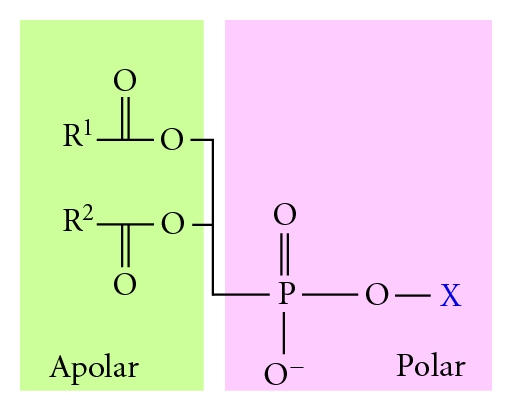
Departmental structure and charge distribution of a typical phosphoglyceride. On the left is the polar phosphoric group esterified to the hydroxyl group of an alcohol. On the right is the apolar aliphatic chains esterified to the central moiety, which is a glycerol.

A typical phospholipid is divided into four sections (Figure 3) [8]:

1. the fatty acid section,
2. a moiety onto which the fatty acids can be attached,
3. a phosphate group,
4. an alcohol attached to the phosphate.

The fatty acid section acts as a hydrophobic fence while the remaining part of the molecule is hydrophilic and can thus interact with the aqueous surrounding of the liposome. The moiety onto which the fatty acids can be attached is usually glycerol but can also be sphingosine [9, 10]. Laboratory liposomal formulation uses glycerol-based phospholipids, also called phosphoglycerides. Glycerol's hydroxyl groups of C1 and C2 are each esterified to the carboxyl group of a fatty acid. The C3 hydroxyl group is esterified to a phosphate group which is in turns esterified to the hydroxyl group of an alcohol. This alcohol group usually belongs to choline, serine, and glycerol but can be also provided from inositol or ethanolamine. Eukaryotes and bacteria membrane lipids are usually phospholipids like phosphatidyl ethanolamine (PA) and phosphatidyl choline (PC).

#### 3.1.2. Lipidic Hydrophobicity

**Figure 4:**
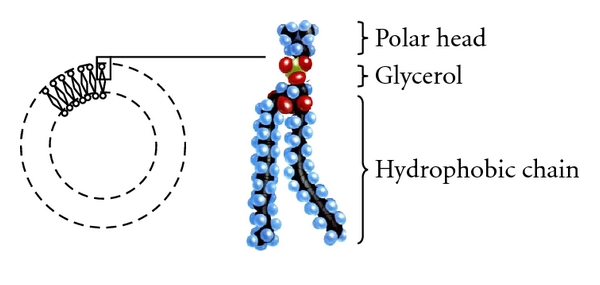
Examination of a lipidic bilayer. The longer apolar chains are stacked in the internal department of the bilayer. Because of their length, they are able to move across the surface of their lipidic sheet, granting the membrane with a valuable fluidity. The darker spots represent the polar heads of each lipid.

It is a fact that lipids have a strong tendency to form membranes. One has to underline that this happens because of their amphipathic nature. On one hand, their polar heads promote aqueous interactions; on the other hand, their long apolar aliphatic chains prefer to interact with each other, stacking themselves side by side. In order to serve both needs, the simplest solution is the formation of a lipid bilayer consisted of two lipid sheets. The hydrophobic chains of each sheet face each other and compose a lipophilic internal compartment that works as a permeability barrier, both inwards and outwards (Figure 4). The forces behind the rapid impulsive formation of these double lipid sheets are hydrophobic interactions. They lower the system's energy by enveloping the aliphatic chains and placing them next to each other thus extruding any water molecules that are unfavourably surrounding hydrophobic regions. Van der Waals forces strengthen this architecture by keeping together the long hydrocarbon tails. Finally, polar interactions and hydrogen bonds generated between the polar heads of lipids and water molecules of the aqueous environment confirm this organization [11, 12].

#### 3.1.3. Lipophilic Permeability Barrier

Because lipid bilayers are put together mainly by hydrophobic forces, these have important biological effects on them:

- lipid bilayers have an innate disposition to increase their surface;
- at some point, lipid bilayers will try to close themselves so that there are no loose ends with hydrophobic chains in direct contact with water molecules, hence forming liposomes;
- in case of a lipidic gap, bilayers maintain themselves by filling it since a breach in the membrane is energetically unfavourable.

Studies carried on the permeability of lipidic membranes show that these bilayer membranes have a very low permeability for ions and almost all polar molecules [13]. Water molecules however are noted to traverse the membranes quite easily, perhaps due to their small size, high concentration, and absence of net charge. Moreover, there is a great variation to the permeation speed among different molecules. For instance, tryptophan (a zwitterion at pH 7) crosses the membrane 1000 times as slowly as does indole, a molecule with a very similar structure, only neutral. Actually, the permeability coefficient of small molecules corresponds to the difficulty these molecules face when they attempt to shed their aqueous cover and adopt a lipophilic one. The less energetically unfavourable this change is, the more easily a molecule can traverse the membrane.

#### 3.1.4. Chemical Instability

**Figure 5:**
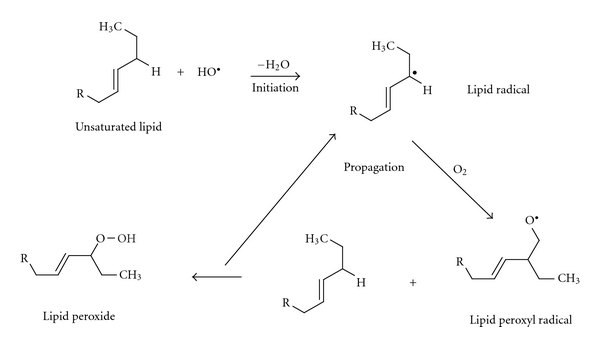
The process of lipidic peroxidation.

Liposomes can be chemically degraded either because of hydrolysis of their ester groups or because of lipid peroxidation (Figure 5). Hydrolysis depends on temperature, pH, and the configuration of lipids in the membrane [14]. Peroxidation occurs when the environment of lipids contains highly active regents such as heavy metals. Adjusting the pH at 6.5, keeping the temperature low, and protecting liposomes in an inert atmosphere should prevent chemical degradation [3, 8].

### 3.2. Physical Traits

#### 3.2.1. Lipid Bilayer Phase Behavior

One of the most significant properties of any kind of lipids is the effect of temperature on their mobility. This response to temperature changes is known as the phase behaviour of lipids. When temperature rises, lipids pass from the solid phase to liquid phase. The temperature at which lipids reversibly transition from one phase to another is called transition temperature. There is also an intermediate phase, interposed between solid and liquid phase. The main difference between the two extreme phases is the freedom lipids may have to diffuse within the layer: trading places with by-standing lipids and travelling along the lipid bilayer only occur in liquid phase. However, a lipid rarely relocates from the lipidic sheet it belongs to the opposing one, since it is difficult for its polar head to traverse through the hydrophobic core of the bilayer [15].

#### 3.2.2. Stability of Lipidic Membranes

The stability of lipidic membranes is affected by plenty variables. Firstly, liposomes gain mobility as temperature rises and that greatly influences the stability of their membrane, given that it is more penetrable and sensitive when being in liquid phase. Therefore, the encapsulation properties of the liposomes vary along with temperature fluctuations [16]. Also, the ingredients of a liposomal membrane can affect its stability. When it comes to phospholipidic liposomes, the use of an auxiliary molecule like cholesterol is highly advised as it has been shown to rigidify the membrane. The presence of polymers like polyethylene glycol (PEG) prevents them from aggregating and fusing, among other things [3, 17]. Finally, the lipidic polar head that is exposed to the aqueous medium surrounding the liposomes can be subjected to pH changes that potentially alter its charge and hydration state [18].

#### 3.2.3. Size Dispersion

Liposomes are vesicles featuring a diameter of a few dozens to a few thousands nanometers. Since they can incorporate one or more phospholipidic bilayers separated by one or more internal aqueous departments, it is easily understood that the more concentric bilayers exist, the greater the diameter of the liposome will be. Liposomes are differentiated according to their size into “Multilamellar Large Vesicles” (MLVs), “Small Unilamellar Vesicles” (SUVs) of 40 to 100 nm of diameter, “Large Unilamellar Vesicles” (LUVs) of 100 to 500 nm of diameter, and “Giant Unilamellar Vesicles” (GUVs) of 500 to 100 *μ*m of diameter [3, 8, 19].

#### 3.2.4. Net Surface Charge

In general, the liposomal membranes can appear to be overall neutral, positively or negatively charged. Their net charge affects their behaviour in vivo. More particularly, negatively charged liposomes circulate for a shorter time in the blood stream compared to the neutral ones. Furthermore, those that are positively charged appear to be more toxic [4, 9]. However, experiments that involved the formulation of a negatively and a positively charged batch of liposomal amphotericin-B returned comparable tissue distribution values for both groups [20].

#### 3.2.5. Colloidal Instability

**Figure 6:**
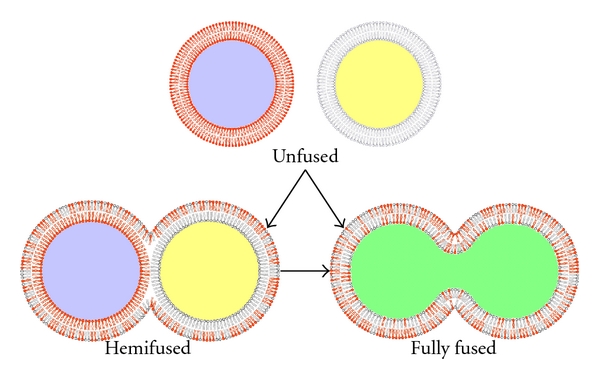
Colloidal instability of liposomes. When two or more liposomes come close enough, their lipidic membranes can interact in two different ways: they can either merge only with their outer lipid sheet (bottom left) or can fuse their entire membranes, essentially blending their contents (bottom right) [6].

In case electrostatic interactions, steric properties, and hydration forces that exist between liposomes allow it, then they have a tendency to aggregate and fuse. Consequently, it depends on the resultant of the aforementioned forces to keep the membranes of liposomes from pressing against each other and potentially merging (Figure 6) [3].

### 3.3. Pharmacokinetics of Liposomes

#### 3.3.1. Pharmacokinetics Overview

Pharmacokinetics of liposomes focuses on their distribution throughout the body fluids and tissues, their metabolism, which mainly includes their chemical degradation, and their excretion, which basically copes with the uptake of liposomes by the mononuclear phagocytic system (MPS) and their clearance. One important goal reached by liposomal formulation is the alteration of the pharmacokinetic profiles of drugs [21]. When agents are carried within liposomes, they actually adopt their carrier's pharmacokinetic disposition until the moment they are released from them. As a result, liposomes have the ability to change both the tissue distribution and the rate of clearance of the drug they carry. Apparently, liberation of the drug from its transporter is required in order for it to exert any therapeutic action. Any drug molecules trapped inside the liposomal core or otherwise still connected to that moiety are practically inert [22, 23].

 The pharmacokinetic behaviour of liposomes is mainly determined by their physiochemical properties along with a series of various other factors. Their physical and chemical traits have been reviewed in a previous section. Hereinafter follows a brief presentation of factors that relate to their administration, metabolism, and release of the active ingredient. One should highlight though that the exact mechanisms through which these parameters act upon and determine the final pharmacokinetic profile of each drug are yet to be fully understood. Producing accurate PK models for liposomes and the drugs they carry is a strenuous and time-demanding procedure. The total concentration of a medicine in the plasma is found under three different forms: the free-form medicine which is the drug molecules that have escaped their liposomal carriers, the liposomal form, which is the concentration of drug still trapped inside the liposomes, and, finally, the plasma protein-covered medicine [3, 24]. The proportion at which these forms coexist in vivo at any given time depends on the stability of liposomal carriers and the nature of the encapsulated molecule. However, we are only interested in monitoring the biopharmaceutical behaviour of the free-form medicine that derives directly from the liposomes. Sadly, that is not always simple or even feasible, either because we cannot distinguish the origin of each form or because we cannot monitor them in the microenvironment of some tumoral sites [25].

#### 3.3.2. Different Administration Routes

Intraperitoneal (I.P.) and intravenous (I.V.) administration tend to have the same effect on mice as well as rats. In I.P. administration, liposomes first enter the lymph and afterwards the blood stream. The only expected difference between I.P. and I.V. introduction of liposomes to the organism is a small lag noticed before the first traces of drug show up in the blood after an I.P. administration.

**In Vivo Distribution of Liposomes**

Once inside the organism, the fate of liposomes depends on their stability in the blood stream as well as their ability to enter various tissues. The former factor is affected by the liposomes quantitative and qualitative composition and their size. Moreover, in the blood stream liposomes interact with plasma proteins, such as opsonins and high- and low-density lipoproteins (HDLs and LDLs, resp.). Opsonins help MPS recognize and eliminate liposomes while HDL and LDL cause rearrangements on their outer lipid layer. These rearrangements usually lead to a rapid release of the enclosed drug to the plasma [26].

#### 3.3.3. Elimination of Liposomes

Elimination of liposomes takes place through three different routes. The first one involves the capture and elimination of liposomes by the MPS. This event results in the excretion of the active ingredient at a hepatic level and its subsequent metabolism, although a small portion of it could reenter the blood circulation. The second elimination route deals with the escape of the active ingredient from circulating liposomes which inevitably leads to greater tissue distribution, metabolism, and excretion for the drug. Finally, there is the passage of liposomes to the tumoral tissues and their local metabolic elimination, a route which is the least favoured of all [27].

#### 3.3.4. Hepatic Adventures

The absorption of plasma proteins on the membrane surface of liposomes causes their opsonization that consecutively leads to their recognition by the MPS, to their accumulation to the liver, and finally to their metabolism by specialized MPS cells called Kuppfer cells. In addition, liposomes are metabolized by splenic macrophages [28].

#### 3.3.5. Release of the Active Ingredient

The kinetics according to which the encapsulated active ingredient traverses the lipid bilayer and exits from its liposomal container depend on the nature of the lipidic membrane and its interactions with the encapsulated drug. The mechanism of release can be described in three steps: the absorption of the active ingredient in the inner lipid layer of the liposome, its diffusion through the membrane, and, finally, its excretion to the surrounding environment. The efficacy of this mechanism depends on the fluidity of the lipid bilayer and on the kind of encapsulated molecule (size, partition coefficient in oil/water).

#### 3.3.6. Delivery of the Active Ingredient to Target Cells

The delivery of the active ingredient requires an interaction between the carrier of the drug and the target cell. Of course, whether the drug is required to be released to the extracellular fluid (ECF) or inside the targeted cell depends on the mechanism of release and the architecture of the carrier [29, 30]. Generally, there are five distinct ways that lead to the liberation of the drug and its disposition to the target cell [3, 31].

1. The first way involves the absorption of liposomes on the membranes of cells where usually after a long period of time, the lipid bilayer of the carrier is degraded by factors like enzymes, lipases, or mechanical strain. That results in the liberation of the active ingredient to the ECF, where they can be diffused towards the cytoplasm—a process that can be especially problematic for hydrophilic molecules. It should be pointed out that once the drug has left its carrier, its pharmacokinetic disposition is the same as to that of the free-form drug.
2. A more straight-forward approach involves the fusion of the liposomal membrane with that of the target cell, hence causing the liberation of the entire liposomal content directly into the cytoplasm.
3. The third and most frequent way is that of endocytosis effectuated by a receptor. This process only regards vesicles of a maximum diameter of 150 nm and active ingredients that can withstand the acidic environment of the lysosomes, the organelles where after the endocytosis liposomes are enzymically processed.
4. The means of phagocytosis concerns liposomes of a size superior to 150 nm and is accomplished by specific cells of the immune system, such as monocytes, macrophages, and Kuppfer cells.
5. Finally, the fifth way involves the transfer of a usually lipophilic active ingredient from the liposomes to the phospholipidic plasma lipoproteins because of the great affinity between the former's bilayer with latter's monolayer.

### 3.4. Liposomes as Drug Carrier Systems

**Figure 7:**
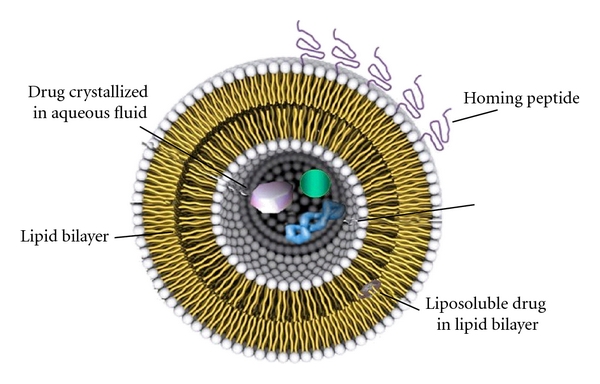
Liposome modulated for drug delivery. In this graphic representation, apart from the basic liposomal structure, one can also observe the protective polymers grafted to the lipid bilayer (upper left), peptides conjugated with lipids for directing the liposome to a certain target (right), a lipophilic molecule soluble in the lipid bilayer, and polar content soluble in the aqueous core.

Significant progress has been achieved in the development and use of carrier vehicles delivering pharmacologically active molecules to tumoral sites over the past decade. Nowadays, the main types of carrier-mediated anticancer agents are indeed liposomes; they are used either alone or conjugated with certain other agents. In theory, the assets of liposome-mediated drugs are greater solubility, longer circulation times, greater exposure, and focused delivery for the enclosed drug (Figure 7). They also provide better therapeutic index and the possibility to bypass resistance related to the free-form of drugs [32]. Hereinafter follows a presentation of the various types of liposomes that have been engineered since that first became feasible, at around 1980. The way liposomes addressed the problems of classic chemotherapy and how scientists handled the drawbacks and limitations of each type of liposomes is also reviewed.

#### 3.4.1. Conventional Liposomes

Conventional liposomes consist of phospholipids like phosphatidylcholine (PC), phosphatidylserine (PS), phosphatidylglycerol (PG), and cardiolipin (CL) [2]. Liposomal formulation of various active molecules handles the two major complications of chemotherapy: limited biodistribution and lack of specified targeting. Liposomes manage to protect the encapsulated molecules from all types of rapid degradation (of enzymatic, immunological, and chemical kind) and passively target tissues or organs that do not have a continuous endothelium, like the liver, spleen, and bone marrow [4]. Another great achievement was the undeniable increase in the solubility of many amphiphilic and lipophilic antineoplastic agents, such as 5-FU. Finally, clinical trials have showcased that liposomal formulations reduce the span of side effects for certain drugs, as in the case of doxorubicin where a lower risk of cardiotoxicity was noted [30, 33].

#### 3.4.2. Pharmacological Impairments

Conventional liposomes were a breakthrough in biomedical technology and still hold a lot of promises for the future. However, this first generation has presented various problems and pharmacological implications over the years. A profound comprehension of these drawbacks allows researchers to tone them down or even bypass them, thus creating more and more effective types of liposomes.

 A main drawback of conventional liposomes is their quick capture by the MPS [34]. Occasionally, this is a desired outcome: high concentration of specific drugs in the MPS can help battle local infections. In most cases, however, the capture of liposomes by the MPS and their removal from blood circulation hampers their therapeutic effect [3, 4]. For the uptake of liposomes by MPS the binding of serum proteins to their surface is required. These are called opsonins and include various protein types, like immunoglobulins and fibronectin among others. A chemical signal for MPS uptake is the complement components as well. Complement system is part of the innate immune system of an organism that directly deals with every substance regarded as a pathogen. It also incorporates various proteins and acts through commencing membrane lysis and facilitating liposomal uptake by the MPS. Surprisingly, the discovery of certain serum components called dysopsonins brought into the light an unexpected ally in the battle against MPS. Human serum albumin and Immunoglobulin A (IgA) actually constraint liposomal recognition and phagocytosis [4].

#### 3.4.3. HDL and LDL

Blood circulating HDL and LDL interact with liposomes and reduce their stability. Lipid transfers and rearrangements take place on the liposomal surface, frequently resulting in lipid depletion, generalized disintegration of liposomes, and loss of the encapsulated active ingredient [3, 7].

#### 3.4.4. The Art of Problem Solving

**Figure 8:**
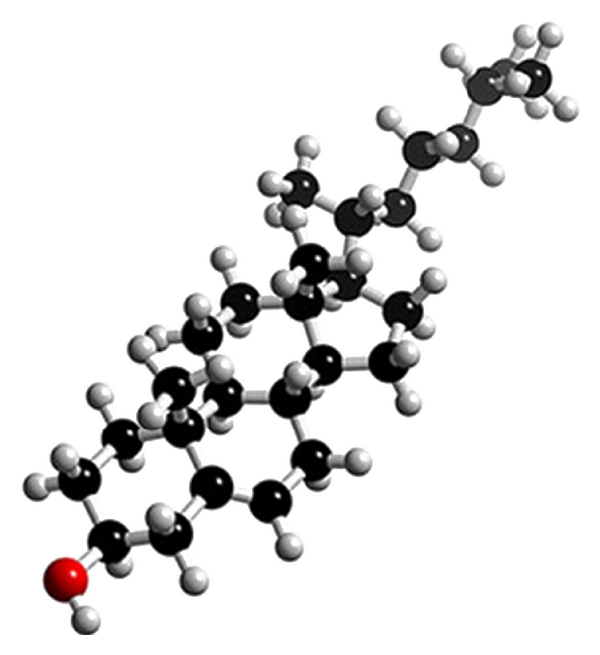
Cholesterol.

In their efforts to address the aforementioned problems, scientists came up with various techniques. Cholesterol (CH) is a hydrophobic molecule and is therefore directed at the core of the membrane, reinforcing it (Figure 8) [16]. The denser packing of phospholipids caused by an excess of cholesterol (more 30%) effectively reduced their transfer to HDL and LDL. So, the addition of CH in the lipidic bilayer lowered their permeability and increased their in vivo and in vitro stability. Moreover, the use of PC with saturated fatty acyl chains and materials that stretch transition temperature beyond 37°C [3] offered an even greater stabilization. Experimenting with variations in liposomal size, researchers also observed that liposomes smaller than 100 nm in diameter (i.e., SUVs) interacted less with plasma proteins, evaded capture by the (MPS), had a greater circulation time, and accumulated passively at the tumoral site eventually. Conversely, it was found that larger liposomes (i.e., MLVs) were eliminated more rapidly from blood circulation and did not manage to avoid MPS uptake for as long as SUVs did. However, a small size also involved reduced storage capabilities. So, a size of 80 to 200 nm is nowadays considered to combine satisfactory reservoir capacities without sacrificing bioavailability.

#### 3.4.5. Long-Circulating Liposomes

Despite all hopes placed on conventional liposomes, they too were finally opsonized and thus recognized by the MPS. Because of a great blood supply and large population of local phagocytic cells, the organs in which they accumulate are the liver and spleen [30]. Mindful that hepatic and spleen accumulation of liposomes is usually an unwanted outcome that also included depletion and modification of the inherent defensive properties of phagocytes, scientists embarked on rendering the liposomes invisible to macrophages [35]. The concept of steric repulsion against opsonizing proteins by placing hydrophilic polymers (i.e., PEG) on the membrane surface of liposomes yields a new category of liposomes called “long-circulating liposomes”, also known as “sterically stabilized liposomes” (SSLs). SSLs are essentially an evolution of conventional liposomes: they are based on the same theory of carrier-mediated drug delivery and feature some characteristics proven to be indispensable, like high CH concentration (40–50% mol/mol) and usually hydrogenated phospholipids [24, 36].

**(a) Polyethylene Glycol**

**Figure 9:**
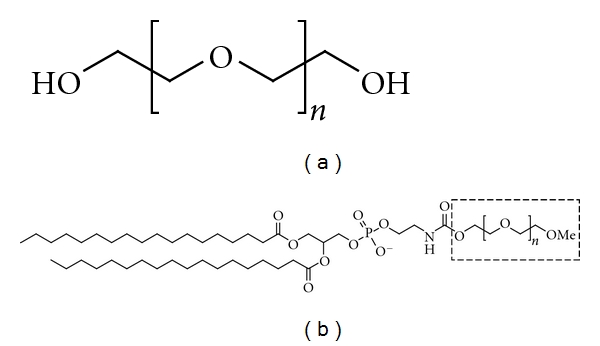
The molecular type of polyethylene glycol (a) and chemical structure of DSPE-PEG (b).

**Figure 10:**
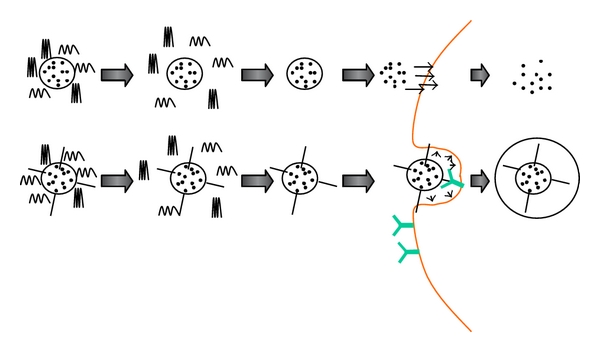
Graphic representation of the two main shedding concepts: (a) the enclosed drug is released to the ECF after PEG polymers have unmasked the liposome upon arrival to the tumoral site and (b) targeting ligands are revealed after their polymer coating has been shed at the moment liposomes arrive to their target; now they are able to interact with appropriate formations on the cell's surface.

PEG (CAS number 25322-68-3) is a linear polyether diol. Its properties include good biocompatibility, high solubility both in aqueous and organic solutions, absence of toxicity, and very low stimulation of the immune system (Figure 9(a)) [37, 38]. PEG polymeric chains are flexible and have been noted to extend approximately 5 nm from the liposomal surface [23], although this value may be increased up to 10% of the diameter of the liposome. In addition, the chains can be easily modified, lengthwise and weightwise. Modification of liposomes with PEG can take place through three different methods. Firstly, the membrane can physically absorb the polymer and place it among its lipidic contents on account of its long apolar chains. Secondly, provided that the polymer is present during the formulation process, it can be incorporated in the newly formed membrane. Finally, PEG can be anchored in the lipidic bilayer by a cross-linked lipid (a covalently attached polymer chain to the amine group of a phosphoglyceride), like PEG-distearoylphosphatidylethanolamine (DSPE-PEG). “Polymer Brush” is characterized by an extensive structure that reaches up to 50 Å from the liposomal surface. “Polymer Mushroom” is a bit more compact form, while “Polymer Pancake” occupies the least space (15 Å) with a structure that lays firmly on the liposomal surface. The various proposed distributions of PEG chains in the lipidic membrane and the space they occupy are represented in Figure 10. PEG chains bring upon plenty enhancements for liposomes. Because of steric repulsion, which also applies between liposomes, PEG promotes their colloidal stability by inhibiting their aggregation and fusion. Most importantly, though, this polymer effectively reduces MPS uptake of liposomes, potentiating all of their related biopharmaceutical properties [39].

**(b) PEG-Coated Liposomes and MPS Uptake**

PEG chains compose a protective, hydrophilic film on the liposomal membrane. Its presence acts as a spatial barrier for other molecules that approach liposomes, like various serum components. Because of the steric hindrance induced by the polymer film, interactions between liposomes and plasma proteins are inhibited. In other words, liposomes are not opsonized nor affected by complement components and consequently evade capture by MPS cells. Interestingly, the ability of PEG to ameliorate liposomal circulation time is usually proportionate to its amount and molecular weight or length [40].

The practical outcome of these phenomena is obvious when comparing the biopharmaceutical profiles of SSLs and conventional liposomes: SSLs have a

- longer half-life (which leads to longer blood circulation times),
- improved tissue distribution (they are avoiding accumulation in healthy tissues),
- low systemic plasma clearance,
- low volume of distribution and,
- multiple-fold greater Area Under the Curve (AUC) values [30].

Summarizing, SSLs have PEG polymeric chains surrounding their outer lipidic sheet and are rich in CH content (40–50% mol/mol) and hydrogenated phospholipids [24]. Their use has allowed encapsulated agents to present an improved therapeutic index. This occurs because of the advanced characteristics of SSLs that permit them to circulate longer in the blood stream, in conjunction with the clearly augmented permeability of the tumoral vascular system [41].

#### 3.4.6. Enhanced Permeation and Retention Effect and Passive Targeting

The phenomenon of Enhanced Permeation and Retention (EPR) effect describes the property of malignant tissue to permit vesicles of proper size to traverse through their vessel walls and enter the tumoral site. Pathological tissues, such as inflammatory or solid tumour tissues, are described by an increased vascular permeability [30, 42]. More particularly, solid tumours that undergo angiogenesis develop discontinuous endothelium. Onto it, large fenestrations allow molecules of up to approximately 4000 kDa or 500 nm to enter the interstitial space. SSLs fulfil the size requirements needed to permeate the tumor vessels and gather in the desired tissue. This phenomenon is called passive targeting and involves the extravasation of liposomes on account for their mechanical and physical properties. More importantly, once liposomes have entered the tumoral tissue, its malfunctioning lymphatic system is incapable of removing them, so they are accumulated in this area, granting time to the encapsulated liposomal load to exert its therapeutic effect [4].

#### 3.4.7. Active Liposomes

Liposomes that can selectively recognize antigens or receptors situated on the surface of specific cells are called “active liposomes” or “3rd generation liposomes”. Sometimes tumour cells express moieties of proteinic nature such as special receptors, transfer systems, and other molecules on their membrane surfaces. If these formations are within reach from the ECF, then specified recognition of cancerous cells is possible by mating medicines with ligands of great affinity to the surface of targeted cells. Given that there will be high selectivity towards the cancerous cells, almost the entirety of administered drug will be accumulated at the tumoral site, leaving nearly intact other healthy by-standing cells. That way, the demanded dose for the same cytotoxic result will be significantly smaller when compared to nontargeted therapies. Practically, this provides for a better therapeutic index with higher drug efficacy and minimized adverse effects. Ligands appropriate for tumor targeting are monoclonal antibodies (MAbs), peptides, carbohydrates, glycoproteins, receptor ligands, and growth factors [43–45].

**(a) Steady, Aim, and Fire: Monoclonal Antibodies and Immunoliposomes**

The mechanism of therapies based on the use of MAb is bimodal. On one hand, MAb can act directly upon specific receptors, stimulating or inhibiting them. These receptors usually cause signal cascades or inhibit certain metabolic paths, either way leading to cell apoptosis. On the other hand, a MAb can intervene and facilitate an immunogenic response of the organism against cancerous cells through IgG mediated procedures, like complement-, antibody-, and cell-mediated cytotoxicity. Unfortunately, the aforementioned technology has a major disadvantage. When using free-form medicines, the more drug molecules are mated with a MAb, the more sensitive the immunologic system becomes against them. The solution to this mishap may come once again from the field of liposomes [46]. The coupling of liposomal carriers with MAb capable of recognizing target cells directs the liposomes to the tumoral site, binds them on the surface of the diseased cells, and helps introduce either the liposomes or the enclosed drug to their cytoplasm. When it comes to using antagonistic antibodies, one can either “plant” them in the lipid bilayer of liposomes or position them at one end of the PEG chains—this last option presenting the benefit of stealth, as well. The resulting liposomes are called immunoliposomes and have been put to the test giving very encouraging results. Of course, the risk of immunoliposomes to be discovered by an organism's immune system still remains. Therefore, in order for them to function effectively, the amount of antibodies placed on their surface must be enough for them to bind to target cells but should not reach the level at which their camouflage is compromised. Immunoliposomes have proven to be quite potent and indeed present a wide array of advantages, especially when compared to targeted free-form medicines [23, 47].

- One can regulate the amount of ligand molecules positioned on the liposomal surface thus pitching the size of uptake.
- They increase the quantity of drug that can be transferred to the target.
- They present the “by-standing killing” effect, meaning that drug molecules can diffuse towards nearby cells and express their cytotoxic action there as well.
- Reduced interactions with nontargeted elements give immunoliposomes a favourable pharmacokinetic behavior.
- Precise action on targeted cells ensues drug efficacy and safety.
- Finally, immunoliposomes are characterized by all properties that make liposomal carriers ideal vectors: they protect their content and do not easily arouse immunologic response from the receiver organism.

**(b) Phage-Liposomes**

Because of the technical limitations one may encounter when anchoring MAbs on liposome (e.g., yield, cost effectiveness) and the potentially limited stability of the resulting immunoliposomes, novel alternative approaches for ensuring better targeting towards specific tissues are being developed. Integration of phage display technology is a promising strategy to achieve selective delivery of liposomes to tumors. Phage display has allowed identification of tumor-specific peptides, but as for immunoliposomes, preparation of phage peptide-targeted liposomes has some serious technical limitations [48]. Using landscape phage fusion coat proteins has being recently proposed as targeting ligands for liposomes. The property of phage coat protein to insert spontaneously into liposome bilayer can be used to generate easily phage-liposomes, thus skipping the conjugation process [49]. Because selection of tumor-binding ligands with phage display libraries is an increasing and promising technology, developing phage-liposomes should in the near future offer challenging opportunities to improve delivery of anticancer drugs entrapped in nanocarriers [50].

#### 3.4.8. Sheddable Coatings

As mentioned before, steric stabilization of liposomes is an indispensable method for achieving longer circulation times and giving the carrier the opportunity to extravasate to the tumoral site. Moreover, ligands with high affinity for formations located on the membrane of cancerous cells offer high selectivity towards these cells, potentially ameliorating the drugs' therapeutic index [51, 52]. However, there is room for improvement in both techniques. On one hand, steric stabilization can sometimes render the liposomal bubble to be too rigid. So, even though liposomes will withstand circulation strains and have enough time to accumulate at the tumoral site, they will not be able to loosen their structure and allow the entrapped drug to exit. On the other hand, once ligands used for targeting therapies, evasive against the immune system though they are, are spotted, the entire therapy is rendered useless [53–55]. A proposed solution for both sterically stabilized and active liposomes can be sheddable coatings.

 Sheddable coatings include molecules for covering the entire liposomes, ligated to the liposomal surface in a reversible way. In the case of SSL, the coating that needs to be shed is apparently the PEG polymer chains. The shedding should take place in the desired point in time, which is right after liposomes have gathered in the malignant tissue. Then, according to one of the techniques that will be presented later, PEG chains unmask the liposomes, who are left to diffuse their content in the ECF fluid. As far as targeted liposomes are considered, their targeting ligands could be covered by an appropriate polymer. That way, immunologic response will be hopefully diminished. This approach seems to be hampering the targeting advantages of these moieties since they will be covered underneath a polymer coat. However, this method also ensures that this liposomal mask will be removed the appropriate time, allowing ligands to interact with their targets [47].

**(a) Shedding Techniques**

Shedding by removing link between the stabilizing polymer and its liaison: these are the three main ways to unshed liposomes through linkage removal.

**(a.1) Acid pH-Induced Shedding**

Receptor-mediated endocytosis is a very common process by which liposomes enter the cells. Liposomes are thus introduced to the lysosomal and endolysosomal compartment where pH is around 4.5 and 5.5–6.0, respectively. Cytoplasmic pH could also be more acidic than normal, when it comes to cancerous and inflammatory tissue. If this is the case, then a pH-sensitive linker between the lipids and polymer coating could undergo protonation, hence hydrolyzing and removing the polymer molecule [18].

**(a.2) Reduction-Based Shedding**

Carriers can also be conjugated with coatings via a linkage sensitive to red-ox potential fluctuations. The disulfide bond (–S–S–) is one of these links. This particular bond is reversible but stable in extracellular environments. However, the high intracellular concentration of molecules presenting sulfhydryl groups creates a reductive environment for the disulfide bond. Its reduction could alternatively take place at the cell surface and at the endosomal and/or lysosomal department by local red-ox enzymes [18].

**(a.3) Proteolysis-Generated Shedding**

In case the link between the polymer coat and its anchor is a peptide, then proteolytic enzymes can hydrolyze it, hence freeing the liposome from its cover. This technique can be put in use since proteolytic enzymes are abundant in the ECF of inflammatory and malignant tissues. Of course, proteolytic enzymes are also available intracellularly in organelles like endosomes and lysosomes [47].

**(a.4) Shedding by Self-Degradation of the Coating Polymer**

Instead of breaking the link between the coating and its anchor, a proposed technique involves the full degradation of the polymer. For this to take effect, coatings must be fully biodegradable though still invisible to the immune system. One solution included chemically modified PEG molecules so as to be sensitive in red-ox changes [47, 56].

## 4. Conclusions

Developing innovative delivery strategies for optimizing therapeutics is an ongoing story. Despite constant efforts made by pharmaceutical and biotechnology companies, it is fully acknowledged now that brand-new pharmacological targets will hardly emerge in the future. Consequently, achieving noninferiority during phase-3 clinical trials is now accepted by most regulatory agencies when approving new chemical entities. In this context, looking backwards to old drugs with new delivery systems is more and more seen as a possible alternative to further increase both drug efficacy and tolerance, and in this respect, nanomedicine is a promising new field of investigation [3]. Although being an old concept, moving towards liposome-encapsulated drugs is still considered as one of the most challenging opportunity to optimize clinical outcome in various diseases, with strong expectations in oncology. Because of their tolerability and similarity with natural, biological membranes, liposomes are less likely to be concerned by the issue of long-term accumulation in the body, as for other emerging nano-objects [57]. Recent efforts made to develop targeted liposomes (e.g., immunoliposomes or fusion phage liposomes) will probably yield, in the near future, a new generation of drugs with unique properties enhancing the effectiveness of drug-based therapy.
